# Differential Regulation of Gene Expression of Alveolar Epithelial Cell Markers in Human Lung Adenocarcinoma-Derived A549 Clones

**DOI:** 10.1155/2015/165867

**Published:** 2015-06-17

**Authors:** Hiroshi Kondo, Keiko Miyoshi, Shoji Sakiyama, Akira Tangoku, Takafumi Noma

**Affiliations:** ^1^Department of Molecular Biology, Institute of Health Biosciences, The University of Tokushima Graduate School, 3-18-15 Kuramoto-cho, Tokushima 770-8504, Japan; ^2^Department of Thoracic and Endocrine Surgery and Oncology, Institute of Health Biosciences, The University of Tokushima Graduate School, 3-18-15 Kuramoto-cho, Tokushima 770-8504, Japan

## Abstract

Stem cell therapy appears to be promising for restoring damaged or irreparable lung tissue. However, establishing a simple and reproducible protocol for preparing lung progenitor populations is difficult because the molecular basis for alveolar epithelial cell differentiation is not fully understood. We investigated an *in vitro* system to analyze the regulatory mechanisms of alveolus-specific gene expression using a human alveolar epithelial type II (ATII) cell line, A549. After cloning A549 subpopulations, each clone was classified into five groups according to cell morphology and marker gene expression. Two clones (B7 and H12) were further analyzed. Under serum-free culture conditions, *surfactant protein C* (*SPC*), an ATII marker, was upregulated in both H12 and B7. *Aquaporin 5* (*AQP5*), an ATI marker, was upregulated in H12 and significantly induced in B7. When the RAS/MAPK pathway was inhibited, *SPC* and *thyroid transcription factor-1* (*TTF-1*) expression levels were enhanced. After treatment with dexamethasone (DEX), 8-bromoadenosine 3′5′-cyclic monophosphate (8-Br-cAMP), 3-isobutyl-1-methylxanthine (IBMX), and keratinocyte growth factor (KGF), *surfactant protein B* and *TTF-1* expression levels were enhanced. We found that A549-derived clones have plasticity in gene expression of alveolar epithelial differentiation markers and could be useful in studying ATII maintenance and differentiation.

## 1. Introduction

Lung diseases such as chronic obstructive pulmonary disease and idiopathic pulmonary fibrosis can be life threatening. Until now, lung transplantation has been the treatment of choice for the severe cases [1]. However, lung transplantation is associated with several problems, including issues with histocompatibility and a shortage of donors. Therefore, regenerative medicine of the lungs using stem cells is attracting a lot of attention as a promising therapy [2, 3]. Recently, embryonic stem cells (ESCs) and induced pluripotent stem cells (iPSCs) have been used to study the possible regeneration of alveolar epithelial type (AT) cells [4, 5]. Differentiation into AT cells from ESCs and iPSCs still needs to pass through the several developmental stages, and the regulation of this developmental process remains unclear. Thus, it is required to establish a simple and reproducible model system to understand the molecular basis of the differentiation of divergent progenitor populations in the human lung and to further develop lung regenerative therapy.

Lung alveoli, which are essential for respiratory function, are composed of two types of alveolar epithelial cells, that is, type I (ATI) and type II (ATII). ATI cells are flat cells that cover 95% of alveoli, and they are involved in the exchange oxygen and carbon dioxide [6, 7]. These cells express specific differentiation markers, such as aquaporin 5 (AQP5 [8, 9]), caveolin-1 [10], and the receptor for advanced glycation end products [11]. ATII cells are cuboidal cells and produce surfactant, which consists of proteins such as surfactant proteins A, B, C, and D (SPA, SPB, SPC, and SPD), and phospholipids. These surfactants are essential for maintenance of alveoli and host defense [12–14]. SPA, SPB, and SPD are synthesized in both Clara cells and ATII cells. SPC is synthesized only in ATII cells and, therefore, is a specific marker for ATII cells [15]. The cell-type-specific expressions of SPB and SPC in Clara and ATII cells are required for lung respiratory function [16, 17]. Both gene expressions are regulated by thyroid transcription factor 1 (*TTF-1*) during lung development [18–20]. ATII cells have the stem cell-like properties of self-renewal, proliferation, and differentiation into ATI cells following injury [21–26]. Therefore, it is quite important to prepare a simple and reproducible ATII cell model system and establish a protocol to control the differentiation into ATI cells.

In this study, we used a human non-small cell lung cancer-derived cell line, A549, to explore the possibility whether A549 cells are suitable for investigating the regulation of gene expression of differentiation markers. A549 cells are well studied and known to have both* K-RAS* mutations (G12S) and* epidermal growth factor receptor* (*EGFR*) gene amplification [27–29]. A549 cells retain some of the properties of ATII cells but do not express some genes such as* TTF-1* [30–32]. However, A549 cells have also been reported to have morphological heterogeneity with various proliferative activities [33] and are not sensitive to differentiation stimuli, for example, insulin/dexamethasone (DEX) treatment [34]. Therefore, we first isolated A549 clones and investigated their gene expression patterns in response to several differentiation stimuli. We found that A549 clones responded reproducibly to their stimuli, showing the plasticity in the gene expression of differentiation markers. These findings indicated that A549 clones could be used for an* in vitro* system to study molecular basis of AT cells differentiation.

## 2. Materials and Methods

### 2.1. Cell Cultures

A549 cells, a human non-small cell lung carcinoma cell line, were cultured in Dulbecco's modified Eagle's medium (DMEM, Nissui, Tokyo, Japan) containing 10% fetal bovine serum (FBS, JRH, Bioscience, Lenexa, KS, USA) at 37°C in a 5% CO_2_ incubator. For maintenance, A549 cells were passaged at 70% confluence, and medium was changed every 3 days. Hereafter, the original A549 cells are referred to as parental cells and cloned cells are referred to as “clones” with individual letters and numbers.

### 2.2. Characterization of A549 Clones

#### 2.2.1. Single Cell Cloning

A549 clones were isolated by limiting dilution of A549 cells. Briefly, A549 cells were washed twice with phosphate-buffered saline without calcium and magnesium [PBS(−)] and dissociated with 0.083% trypsin (Sigma-Aldrich, Tokyo, Japan) and 0.177 mM ethylenediaminetetraacetic acid. Cell numbers were counted with trypan blue staining. The cells were seeded at 0.3 or 1 cell/well into two 96-well plates. When each isolated clone was grown to 80% confluence, the cells were sequentially transferred into 24-well plates, 6-well plates, and 6 cm dishes.

#### 2.2.2. Morphological Analysis of A549 Clones

Morphology of A549 cell clones was observed using optical microscope (CKX41N 31PHP; Olympus Corporation, Tokyo, Japan), and the images were captured using a digital microscope camera (DS-Fi2-L3; Nikon, Tokyo, Japan). The thickness of the cells was analyzed by the mean gray value using ImageJ (National Institute of Health, Bethesda, MD, USA) as follows: thin, <5; thick, ≥5. The boundary of cell clusters was evaluated by smoothness of the cell cluster outline as follows: “clear” was a smoothly drawn cluster outline and “unclear” was a hard to draw cluster outline. The cell density was evaluated by cell numbers within the frame of 100 *μ*m × 100 *μ*m at 70% confluence and evaluated as follows: high, ≥10 cells; low, <10 cells. The number of cell clusters was observed as the clustering feature at 1 day after passage using high, ≥3; low, <3.

#### 2.2.3. Gene Expression Analysis

**Figure 1:**
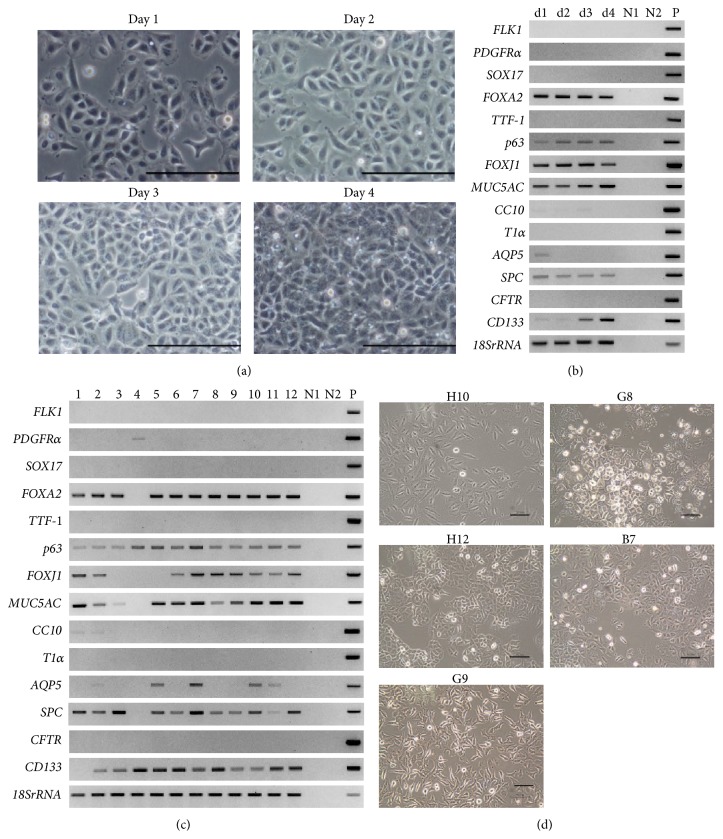
Characterization of A549 cells. (a) Representative morphology of A549 parental cells during growing phase. Cells were monitored for 4 days after passage. Scale bars: 100 *µ*m. (b) Gene expression of A549 parental cells shown in (a).* 18SrRNA* was used as the internal control. d1 to d4: day 1–day 4 shown in (a), N1: negative control of PCR products without RT reaction, N2: negative control with distilled water, and P: positive control with sequenced PCR products in plasmid. (c) Gene expression of A549 clones. The number indicated the group of clones as shown in Table 3. N1, N2, and P are the same symbols as in (b). (d) Representative morphology of A549 clones at 70% confluence. A549 clones were classified into five groups as shown in Table 4 and selected the representative clones. Scale bars: 100 *µ*m.

The levels of gene expression were determined by reverse transcription polymerase chain reaction (RT-PCR). Briefly, total RNA was extracted from the cells using Tri Reagent (Molecular Research Center, Cincinnati, OH, USA) following the manufacturer's protocol. RT was performed using RNA PCR kit AMV ver. 3.0 (TaKaRa, Shiga, Japan). Synthesized cDNA was used for PCR using Go Taq DNA Polymerase (Promega, Madison, WI, USA). Gene-specific primers and PCR conditions are listed in Table 1. To detect* TTF-1* expression, PCR was performed by 30 cycles for the initial characterization of clones (Figure 1 and Table 2) and 40 cycles in the other experiments. To detect* SPC* expression, the primer set of SPC1 was only used for the characterization of clones (Figure 1 and Table 2), and the primer set of SPC2 was used in the other experiments.

#### 2.2.4. Densitometric Analysis

The expression level of each transcript was normalized by that of* 18S ribosomal RNA* (*18SrRNA*). The images of the electropherogram were captured using ChemiDoc XRS (BIO-RAD, Hercules, CA, USA), and volume analysis was performed using Quantity One (BIO-RAD). The relative ratio was calculated by time 0 as 1. In the case of no expression signals at time 0, the relative ratio was calculated by the lowest expression level among the samples as 1.

### 2.3. Cell Proliferation Assay

The proliferative activity of the cells was evaluated by counting cell numbers at the indicated time points using trypan blue and Luna-FL Dual Fluorescence Cell Counter (Logos Biosystems, Inc., Gyunggi-Do, Korea).

### 2.4. Induction of Cell Differentiation

#### 2.4.1. Serum-Free System in Culture

Approximately 4.2 × 10^5^ cells of the A549 cell clones, B7 and H12, were seeded in 6-well plates in DMEM containing 10% FBS. After the cells reached 70% confluence, they were washed twice with PBS(−), and DMEM without FBS was added. Cell numbers were counted at 12, 24, 48, 72, 96, and 120 h. For RT-PCR analysis, the cells were harvested every 24 h.

#### 2.4.2. Stimulation of Epidermal Growth Factor Receptor (EGFR) Signaling Pathway

B7 and H12 were seeded in 6-well plates in DMEM containing 10% FBS. After the cells reached 70% confluence, they were washed twice with PBS(−), changed into DMEM without FBS, and human EGF (10, 30, and 90 ng/mL; Pepro Tec, Rocky Hill, NJ, USA) was added. For RT-PCR analysis, the cells were harvested every 24 h.

#### 2.4.3. Inhibition of RAS/Mitogen-Activated Protein Kinase (MAPK) Signaling Pathway

B7 and H12 were seeded in 6-well plates in DMEM containing 10% FBS. After the cells reached 70% confluence, they were first treated with the selective MEK1/2 inhibitor U0126 (3, 10, and 30 *µ*M; Wako, Osaka, Japan) or the MEK1 inhibitor PD98059 (30, 60, and 90 *µ*M; Wako) in DMEM containing 10% FBS. Cell numbers were counted at 12, 24, and 48 h. For RT-PCR, the cells were treated with U0126 (10 and 30 *µ*M) or PD98059 (90 *µ*M) and were harvested at 24 and 48 h.

#### 2.4.4. Combination with MEK Inhibitor and DEX-[8-BR-cAMP]-IBMX-KGF (DCIK) Treatment

B7 and H12 were seeded into 6-well plates in DMEM containing 10% FBS. After the cells reached 70% confluence, they were first treated with U0126 (30 *µ*M) in DMEM containing 10% FBS. After 24 h, they were washed twice with PBS(−), and the medium was changed to DMEM without FBS ± DEX (50 nM; Wako), 8-Br-cAMP (0.1 mM; Sigma-Aldrich), isobutyl-1-methylxanthine ([IBMX], 0.1 mM; Wako), and keratinocyte growth factor ([KGF], 50 ng/mL; Wako) for 3 days. DCIK control was cells without DCIK. The cells were then washed twice with PBS(−) and cultured in DMEM with or without FBS for an additional 3 days.

### 2.5. Statistics

Statistical analysis was performed by Student's* t*-test using Microsoft Excel software. The* p* value less than 0.05 indicates being statistically significant.

## 3. Results

### 3.1. Characterization of A549 Parental Cells and Clones

To analyze the characteristics of A549 parental cells, we first observed their morphology in the growing phase for 4 days after seeding. As shown in Figure 1(a), we detected several variations of morphologies in A549 parental cells. We concurrently examined the expression patterns of 14 differentiation marker genes using one of the paired experimental sets (Figure 1(b)) and summarized the results in Table 2. We found that the forkhead box genes, that is,* FOXJ1* and* FOXA2*,* transformation-related protein 63* (*p63*),* mucin-5AC* (*MUC5AC*) which is a goblet cell marker, and* SPC* were constantly expressed at the similar levels throughout the culture period. These genes are the specific markers for endoderm cells, basal cells, ciliated cells, goblet cells, and ATII cells, respectively.* Aquaporin 5* (*AQP5*), a marker for ATI cells, was expressed on day 1 only, and* Clara cells 10 kDa secretory protein* (*CC10*), a marker for Clara cells, was slightly expressed on days 1–3 during the growing phase. Expression of* Prominin-1* (*CD133*), a marker for cancer stem cells, was enhanced with the days of culture. To obtain clones from A549 parental cells, we next performed limiting dilution and 46 clones were isolated. We classified these clones into 12 groups according to morphological differences. The average number of days to obtain a confluent monolayer of each clone was 3.7 except for clone G8 cells (14 days). Other morphological characteristics of each clone, including cell thickness, appearance of cell cluster boundaries, cell density, and clustering, were observed and are shown in Table 3. We further examined the expression profiles of marker genes using representative clones from each group (Figure 1(c)). Expressions of* FLK1*,* SOX17*,* TTF-1*,* T1α*, and* CFTR* were not detected in all clones. In contrast, all clones expressed* p63*. Clone G8 in group 4 uniquely expressed the gene encoding* platelet-derived growth factor receptor α* (*PDGFRα*), but did not express* FOXA2*,* MUC5AC*, or* CD133*.* FOXJ1* was not expressed in groups 3, 4, and 5.* CC10* was slightly expressed only in groups 1 and 2.* AQP5* was expressed only in groups 2, 5, 7, 10, and 11, and* SPC* was detected in all of them except group 1.

Based on these data, we further classified them into five classes, as shown in Table 4. The representative cell morphology was demonstrated in Figure 1(d). H10 in class 1 was spindle shaped with a slim nucleus.* MUC5AC*, a goblet cell marker, was highly expressed, but* SPC* and* AQP5* were not detected in H10. G8, in class 2, grew slowly and showed a piled-up growth.* SPC*,* p63*, and* PDGFRα* were expressed, but* FOXA2*,* MUC5AC*, and* CD133* were not detected. H12, in class 3, had a tendency to cluster and the outline of the cell cluster boundary was bright and clearly visible. The cells expressed both* AQP5* and* SPC*, which are typical markers for ATI cells and ATII cells, respectively. B7, in class 4, was polygonal shaped with high cell density but the outline of the cell boundaries was unclear. B7 expressed* SPC*, but not* AQP5*. G9 in class 5 shared similar gene expression patterns with B7 but showed invadopodia-like structures.

The gene expression pattern shown in Table 4 suggested that B7 had ATII-like characteristics, and H12 had both ATI-like and ATII-like characteristics. These clones were used for further analyses as potential representatives of ATII and ATI/ATII, respectively.

### 3.2. Effects of Serum Depletion on B7 and H12 Clones

**Figure 2:**
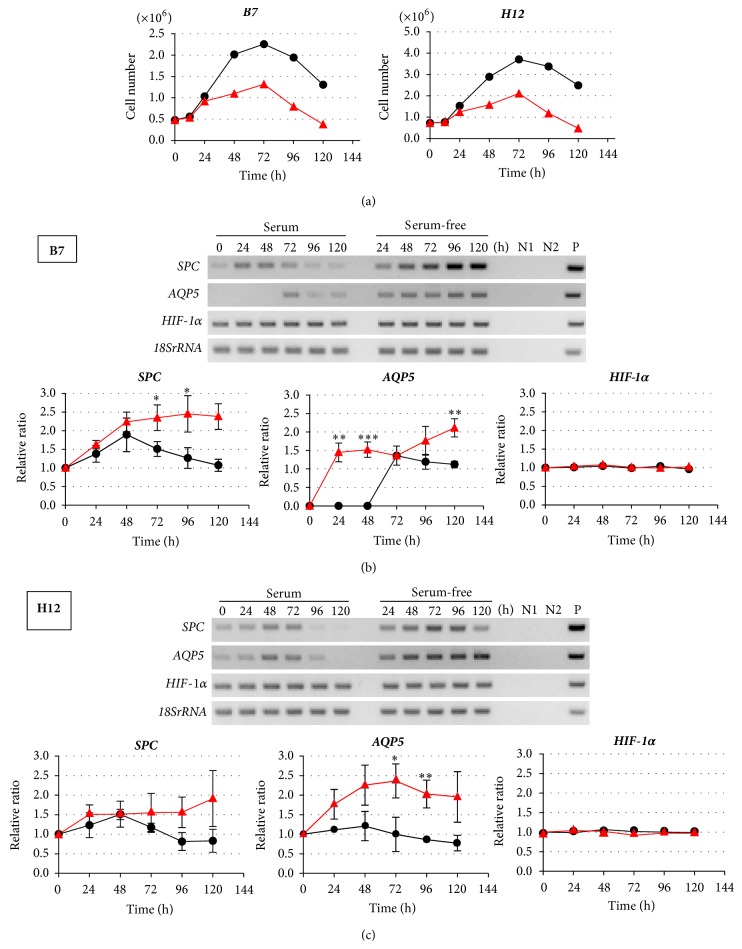
Effects of serum depletion on cell characteristics of A549 clones. (a) Growth curve of B7 (left) and H12 (right). Black: cultured with serum, red: cultured without serum. (b) and (c) Expression and quantitative analyses of* SPC*,* AQP5*, and* HIF-1α* mRNA in B7 cells (b) and in H12 cells (c). (Upper panel) representative RT-PCR results. N1: negative control without RT reaction, N2: negative control with distilled water, and P: positive control with sequenced PCR products in plasmid. (Lower three panels) quantitative analysis of gene expression. Black: cultured with serum, red: cultured without serum. Experiments were independently performed in triplicate. ^*∗*^ *p* < 0.05; ^*∗∗*^ *p* < 0.01; ^*∗∗∗*^ *p* < 0.001.

Proliferation and differentiation are reciprocally and tightly regulated [35]. Because A549 cells are derived from lung adenocarcinomas, it was generally thought that the cells reduced serum dependency and acquired the increase in autonomous growth. Thereby, we initially examined the effect of serum depletion on their proliferation (Figure 2(a)). Both B7 and H12 increased their cell numbers in the culture medium with serum until 72 h. However, cell proliferation was suppressed from 48 h in the serum-free medium. After 72 h, cell numbers started to decrease in both culture conditions. Next, we examined the effects of serum depletion on the gene expression of* SPC*,* AQP5*, and also* hypoxia-inducible factor-1α* (*HIF-1α*) which regulates the phenotypes of cancer cells including their progressive proliferation [36–38] (Figures 2(b) and 2(c)). In B7, significant enhancement of* SPC* expression was observed from 48 h by serum depletion compared with control (Figure 2(b)). At 120 h, the expression level was upregulated 2.9-fold compared with that at time 0. In contrast,* AQP5* was induced significantly at 24 h and the expression level was maintained until 120 h by serum depletion. The expression level of* HIF-1α* was not significantly altered.

On the other hand, in H12,* SPC* expression was enhanced from 24 h by the serum depletion when compared to the control (Figure 2(c)). The maximum expression level was observed at 72 h as a 1.7-fold increase compared with that at time 0. However, in contrast to B7, the expression level started to decrease from 96 h in conditions with or without serum.* AQP5* expression was enhanced in a time-dependent manner by serum depletion, and the maximum expression level was 2.8-fold at 120 h compared with that at time 0.* HIF-1α* expression was not significantly altered. All of these results indicate that the response to serum depletion was different between B7 and H12.

### 3.3. Effects of EGF Treatment on Gene Expression in B7 and H12 Clones

**Figure 3:**
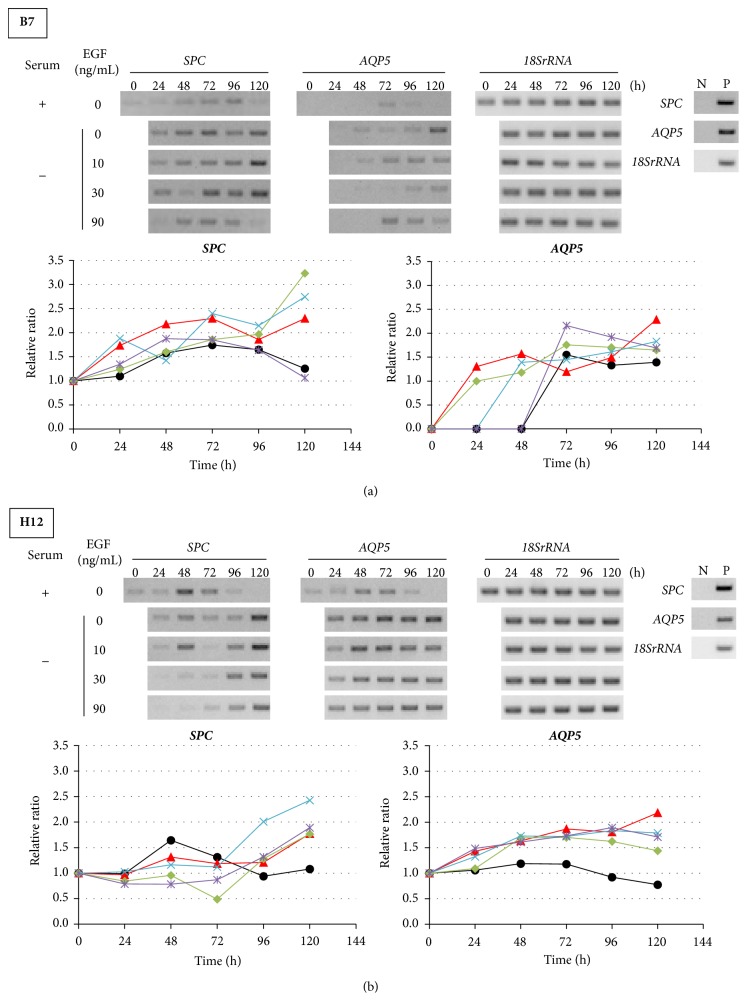
Effects of EGF treatment on gene expression in B7 and H12 clones. (a) and (b) Expression and quantitative analyses of* SPC* and* AQP5* mRNA in (a) B7 and in (b) H12. (Upper panel) representative RT-PCR results. Serum +: cultured with serum, −: cultured without serum, N: negative control without RT reaction, and P: positive control. (Lower panel) quantitative analyses of gene expression. Black: serum, red: serum-free, green: serum-free + EGF 10 ng/mL, light blue: serum free + EGF 30 ng/mL, and purple: serum-free + EGF 90 ng/mL. All data were independently obtained twice.

Because serum contains growth factors and A549 cells have been reported to undergo* EGFR* amplification [29], we hypothesized that the effects of serum depletion could be because of the absence of EGF in the serum. To confirm this possibility, we cultured B7 and H12 in the serum-free medium with EGF (10, 30, and 90 ng/mL). In B7,* SPC* expression was enhanced from 24 h by serum depletion (Figure 3(a)). Ten and 30 ng/mL of EGF treatment did not affect* SPC* expression. A minor suppression of* SPC* was observed by 90 ng/mL of EGF treatment. In contrast,* AQP5* expression was induced from 24 h by serum depletion. By the addition of EGF, the induction of* AQP5* was delayed in a dose-dependent manner. In H12, the enhancement of both* SPC* and* AQP5* expressions by serum depletion was not affected by EGF treatment (Figure 3(b)). These results indicated that EGF in serum-containing medium had little effect on the alveolus-specific gene expression.

### 3.4. Effects of MEK Inhibitors on Gene Expression in B7 and H12 Clones

**Figure 4:**
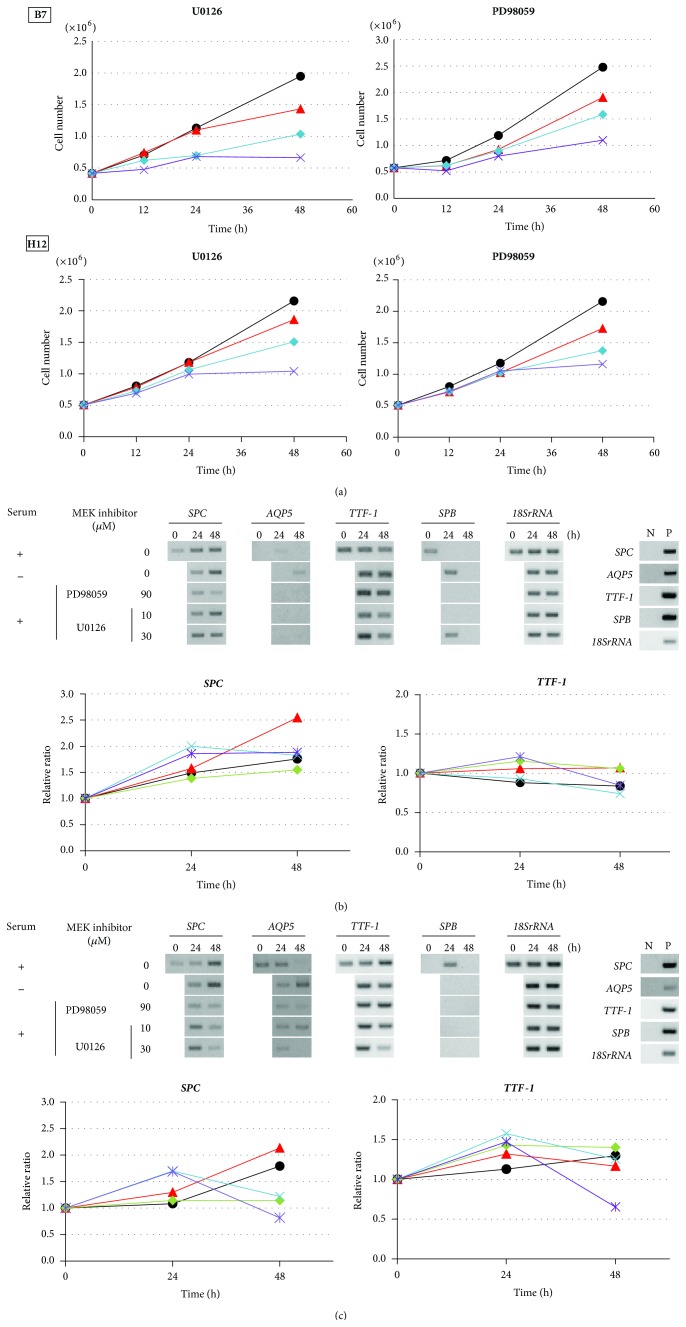
Effects of MEK inhibitors on gene expression in B7 and H12 clones. (a) Growth curve of B7 and H12 in serum-present medium treated with U0126 and PD98059, respectively. U0126 were treated with 3 *μ*M (red), 10 *μ*M (light blue), and 30 *μ*M (purple), and PD98059 were treated with 30 *μ*M (red), 60 *μ*M (light blue), and 90 *μ*M (purple) for the indicated time. Black indicted no treatment with MEK inhibitors. (b) and (c) mRNA expression and quantitative analysis of* SPC*,* AQP5*,* TTF-1*, and* SPB* by RT-PCR in B7 (b) and in H12 (c). Each data was independently obtained twice. (Upper panel) representative RT-PCR results. N: negative control, P: positive control. (Lower panel) quantitative analyses of gene expression. Black: cultured with serum, red: cultured without serum, green: PD98059 90 *μ*M, light blue: U0126 10 *μ*M, and purple: U0126 30 *μ*M.

A549 cells have been demonstrated to have the* K-RAS* mutation (G12S) and show activation of MAPK signaling [27, 28]. To examine whether serum depletion effects could be replaced by inhibition of MAPK signaling, we treated B7 and H12 with U0126 and PD98059. We first confirmed the effects on cell proliferation of both clones treated with U0126 (3, 10, and 30 *μ*M) or PD98059 (30, 60, and 90 *μ*M), respectively (Figure 4(a)). In both B7 and H12, cell proliferation was suppressed by both U0126 and PD98059 in a dose-dependent manner. The most effective dose was 30 *μ*M of U0126 and 90 *μ*M of PD98059. Although these concentrations seemed to be relatively high compared to the dosages used in the previous studies to inhibit ERK phosphorylation (e.g., 10 *μ*M of U0126 and 25 *μ*M of PD98059 [39, 40]), we did not observe increasing numbers of floating cells dependent on the culturing time (data not shown). When we harvested the attached cells, the morphology was the same as that of healthy cells, and the viability of collected cells was more than 90% (data not shown). Therefore, we assessed both concentrations of inhibitors were not toxic. A slight difference in growth response to MEK inhibitor was observed between B7 and H12. B7 was much more sensitive to both inhibitors than H12 and showed earlier suppression of cell proliferation than H12.

Next, we examined the effects of the MEK inhibitors on gene expression in B7 and H12. In B7,* SPC* expression was transiently upregulated 1.9-fold at 24 h by U0126 treatment; however, it was decreased by PD98059 treatment when compared with the control (Figure 4(b)). Although* TTF-1* expression was not detected by 30 cycles, it could be detected by 40 cycles. Under this PCR condition,* TTF-1* expression was upregulated 1.2-fold after U0126 treatment and 1.1-fold after 24 h treatment with PD98059. At 48 h,* TTF-1* expression was decreased under all conditions except serum depletion. The effects of the MEK inhibitor on both* AQP5* and* SPB* expression were varied. On the other hand, in H12,* SPC* expression was transiently upregulated 1.7-fold at 24 h after U0126 treatment, but not after PD98059 treatment (Figure 4(c)).* TTF-1* expression was transiently upregulated 1.5-fold at 24 h after U0126 treatment and 1.4-fold after PD98059 treatment. Expression levels of* AQP5* and* SPB* were not altered by U0126 and PD98059 treatment.

### 3.5. Effects of DCIK Treatment on B7 and H12 Clones

**Figure 5:**
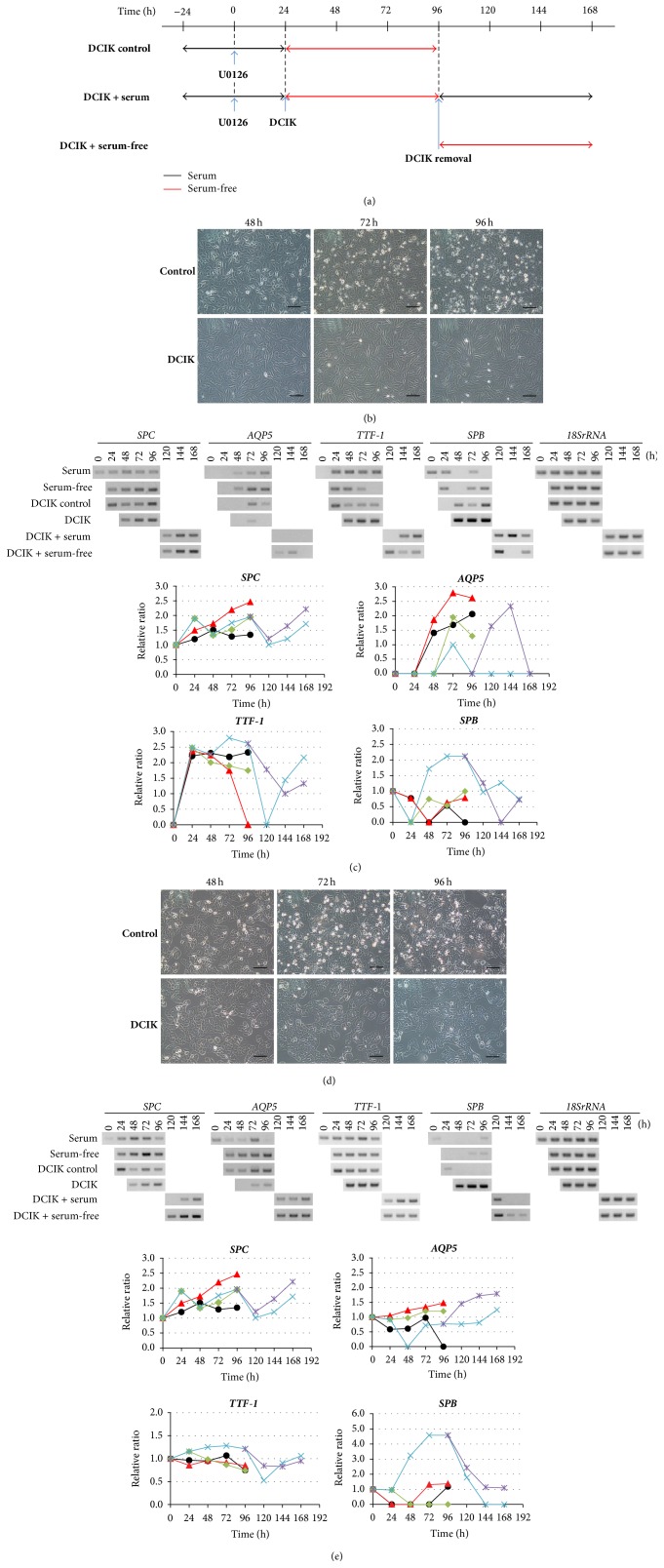
Effects of DCIK treatment following U0126 treatment. (a) Experimental design. B7 and H12 were treated with DCIK for 3 days following U0126 30 *µ*M treatment for 24 h. After DCIK removal, B7 and H12 were cultured with serum or without serum for 3 days. (b) Morphological change of B7 treated without DCIK (control) and with DCIK (DCIK) at every 24 h for 3 days following U0126 treatment for 24 h. Scale bar: 100 *µ*m. (c) Expression and quantitative analysis of* SPC*,* AQP5*,* TTF-1*, and* SPB* mRNAs in B7. (Upper panel) RT-PCR results. (Lower four panels) quantitative analyses of gene expression. Black: cultured with serum, red: cultured without serum, green: cultured without DCIK following U0126 treatment for 24 h, light blue: cultured with DCIK following U0126 treatment for 24 h and cultured with serum following DCIK treatment, and purple: cultured without serum following DCIK treatment. (d) Morphological change of H12 treated without DCIK (control) and with DCIK (DCIK) at every 24 h for 3 days following U0126 treatment for 24 h. Scale bar: 100 *µ*m. (e) Expression and quantitative analysis of* SPC*,* AQP5*,* TTF-1*, and* SPB* mRNAs in H12. (Upper panel) RT-PCR results. (Lower four panels) quantitative analyses of gene expression. Indications were the same as in (c). All data were independently obtained twice.

Previous studies reported that* TTF-1* can promote* SPC* and* SPB* expression [18–20]. Although* SPC* and* TTF-1* expression was enhanced after U0126 treatment was transiently observed in some cases, it was not reproducible in our experiments. Recently, DCIK treatment has been reported to promote differentiation of progenitor cells induced from mouse ESCs and human iPSCs into ATII cells [41, 42]. Furthermore, the tissue-derived ATII cells could maintain their characteristics* in vitro* with DCI treatment [43, 44]; in addition, following DCI removal, they could enhance the expression for ATI cell markers [45]. To test whether A549 clones respond to the culture conditions with DCIK, we next examined the effects of DCIK addition on the expression of ATII cell marker genes, following MEK inhibitor treatment. As shown in Figure 5(a), we first cultured B7 and H12 cells at 70% confluence with 30 *μ*M of U0126 for 24 h. After that, we changed the medium without DCIK (DCIK control) or with DCIK (DCIK) in the serum-free medium and continued to culture for 72 h (total culture time was 96 h). After DCIK treatment, we exchanged the culture medium to remove DCIK and cultured with serum (DCIK + serum) or without serum (DCIK + serum free) for 72 h. The morphology of B7 was significantly changed by DCIK treatment (Figure 5(b)). The shape of B7 became flat and spindle-like after 48 h, and the outline of cell border became clearer. These elongated cells increased in a time-dependent manner, but the clear outline of cells was maintained at the similar level during the entire culture period.

We next examined the effects of combined treatment with MEK inhibitor and DCIK on the gene expression in B7 (Figure 5(c)). When compared with time 0,* SPC* expression was enhanced during U0126 treatment until 24 h. After DCIK addition,* SPC* expression was decreased by 48 h and then increased in a time-dependent manner until 96 h. However, these changes were observed in the same manner in the DCIK control. In contrast, at 72 h,* AQP5* was suppressed 0.5-fold in DCIK, when compared with the DCIK control.* TTF-1* expression was significantly increased up to 1.6-fold after DCIK treatment when compared with the DCIK control.* SPB* was also enhanced in a time-dependent manner from 48 h and reached a 2.1-fold upregulation at 96 h. After DCIK removal,* SPC* expression was decreased by 120 h and then increased in medium with and without serum in a time-dependent manner.* AQP5* was expressed only under serum-free conditions. However,* TTF-1* and* SPB* expression levels were decreased by DCIK removal.

We also performed the same experiments with H12. Morphological changes were not observed in H12 after DCIK treatment (Figure 5(d)), but cells were more clustered after 48 h. We next examined the effects of combined MEK inhibitor-DCIK treatment on the gene expression in H12 (Figure 5(e)).* SPC* expression was enhanced during U0126 treatment until 24 h. After DCIK addition,* SPC* expression was decreased by 48 h and then increased in a time-dependent manner until 96 h.* AQP5* was suppressed in DCIK at 48 h and remained at 0.7-fold after 72 h.* TTF-1* expression was upregulated 1.3-fold at 48 h and remained at that level until 96 h.* SPB* expression was significantly increased by 48 h showing, up to 4.6-fold upregulation at 72 h and remaining at that level until 96 h. After DCIK removal,* SPC* expression was decreased by 120 h and then increased in medium with and without serum in a time-dependent manner.* AQP5* was increased in a time-dependent manner in serum-free culture medium.* TTF-1* decreased transiently at 120 h but recovered to the original expression level.* SPB* decreased but* SPC* increased in a time-dependent manner after DCIK removal.

## 4. Discussion

In this study, we tried to establish a simple and reproducible* in vitro* system that can be used to analyze the molecular mechanisms of lung alveolar epithelial cell differentiation. We isolated A549 clones and characterized them by phenotypic screening using their morphology and gene expression patterns of markers (Tables 2–4). According to the major expression patterns of alveolar cell markers [30, 46], two A549 clones, B7 and H12, were further analyzed as tentative representatives for ATII and ATI/II cells.

We examined whether these clones showed any responses to the differentiation stimuli by inducing several culture conditions with or without serum, DCIK, and MAPK inhibitors. Both B7 and H12 had unique responses to those stimuli, reflecting the characteristics of ATII and ATI/II cells, and these responses were stably and reproducibly observed until passage 122 and passage 120, respectively (data not shown).

The finding that serum-free condition could induce the gene expression of alveolar epithelial differentiation markers in A549 clones is consistent with those of the previous study [47].

Since cell growth and differentiation are reciprocally and tightly regulated [35], we characterized the growth rate with and without serum. We observed that A549 cells were highly proliferative in medium with serum, but serum depletion decreased the growth rate and reciprocally increased the expression of differentiation marker genes. In addition, both SPC and AQP5 proteins were not detectable by western blot analyses in serum depletion system (data not shown). Therefore, the cells can be primed but not fully differentiated in the present condition, suggesting that additional signals or environmental condition might be required to induce the protein expression.

Because* K-RAS* mutation (G12S) and* EGFR* amplification were detected in A549 cells, the effects of serum depletion were first considered to be related to the MAPK signaling cascade which is also known to play an important role in proliferation of cancer [48]. We found that the proliferation of A549 clones could be suppressed by the MEK inhibitor U0126 and showed an enhancement in the expression of the differentiation markers,* SPC* and* TTF-1* (Figure 4). U0126, a MEK1/2 inhibitor [49], caused an enhancement in the* SPC* expression up to 24 h and then caused a reduction or maintenance up to 48 h; however, it did not affect* AQP5* expression. PD98059, a MEK1 inhibitor [50], did not affect either* SPC* or* AQP5* expression. This suggests that RAS-MAPK signals might control the switch between proliferation and differentiation in A549 clones. The differential effects of these inhibitors on marker gene expression might be because of different target molecules, but this remains to be determined.

Second, serum contains a variety of factors, including growth factors such as EGF. Lauand et al. demonstrated that A549 cells amplified EGFR without phosphorylation, and EGF stimulation could activate actin filament organization and cell motility rather than inducing proliferation in A549 cells [51]. However, we observed that EGF had little effect on the expression of differentiation markers (Figure 3). Therefore, it is possible that other ligands in serum, such as tumor necrosis factor-*α*, amphiregulin, and heparin-binding EGF-like growth factor [52–55] could activate EGFR-mediated signals. Further analysis is required to determine the mechanism of the enhancement in differentiation marker gene expression under serum-free condition.

To induce cell differentiation, the control of energy metabolism is another important issue to be discussed. The energy metabolism in A549 cells is regulated by the Warburg effect [56]. Aerobic glycolysis is dominant and mitochondrial oxidative phosphorylation is decreased. The switch in energy metabolism from glycolysis to mitochondrial oxidative phosphorylation has been demonstrated to induce differentiation in cancer cells [57]. That prompted us to examine the effects of glycolysis and/or pyruvate dehydrogenase kinase- (PDK-) inhibitors to increase the flow of carbon metabolites into tricarboxylic acid cycle and switch the differentiation cue. We examined the effects of glycolysis by culture in the specific medium (depleted with L-glutamine, glucose, and sodium pyruvate), the inhibitors of both pyruvate dehydrogenase kinase and lactate dehydrogenase combined with U0126 on the gene expression levels of differentiation markers. However, we did not detect any induction or enhancement in gene expression as a result of these treatments (data not shown), suggesting that in our experimental condition, at least in part, glucose metabolism did not have a major role in the A549 clones.

Recently, several reports have studied the suitable culture conditions to maintain ATII function and induce ATI differentiation [41–45]. DCI treatment* in vitro* could maintain and enhance ATII characteristics of natural ATII cells isolated from lung in human fetuses and adults [43, 44]. Furthermore, KGF treatment could increase surfactant protein gene expression and decrease* AQP5* expression [58–60]. Using mouse ESCs and human iPSCs, it has also been reported that KGF combined with DCI treatment leads to the maturation of ATII-like cells from the progenitor cells [41, 42, 61]. Since mouse epiblast stem cells and human iPSCs have been shown to have a similar energy metabolism to cancer cells [62], we used the combination of U0126 and DCIK to suppress cell proliferation and examined the cell morphology and gene expression of alveolar differentiation markers (Figure 5). This treatment induced a morphological change (flat and spindle shape) in B7, and increased cell-clustering in H12, respectively.* SPC* was not significantly increased by DCIK treatment, but* SPB* and* TTF-1* were significantly enhanced.* AQP5* expression, however, was decreased in both clones. These findings suggested that the expression of ATII-marker genes was enhanced by DCIK treatment. The morphological change in B7 which we observed was slightly similar to the epithelial mesenchymal transition; however, epithelial markers* SPB* and* SPC* were detected. Further analysis is required for better understanding the meaning of morphological change induced by DCIK treatment. In addition, ATII-like cells could be differentiated from the isolated ATII cells of human fetal lung, which were maintained in a DCI-added medium, into ATI-like cells, by the removal of DCI [45]. In our system using A549 clones, DCIK removal did not affect either* AQP5* or* SPC* expression but decreased* SPB* expression. These data suggest that A549 clones require the additional cues for ATI induction.

For the* in vivo* events following lung injury, ATII cells proliferate and spread, and their daughter cells could differentiate into ATI cells [21–24]. An* in vitro* study demonstrated that siRNA of transforming growth factor-*β* (TGF-*β*) and recombinant human bone morphogenetic protein- (rhBMP-) 4 could upregulate expression of ATII markers, whereas siRNA of BMP receptors and rhTGF-*β* upregulated expression of ATI markers in mouse ATII cells [63]. Additionally, ATII cells treated with insulin-like growth factor-I (IGF-I) differentiate into ATI-like cells by activation of Wnt5a in rat ATII cells [64]. These findings indicated that cytokine signals might play the key roles in both genetic and epigenetic programs during lung development.

Finally, we obtained an interesting finding about genetic background of B7 and H12 clones by analyzing the cell authentication. Both B7 and H12 maintain A549 signature, except Y chromosome-loss in H12 (data not shown). Y chromosome instability has been reported in some of human cancer, and it is thought that Y chromosome loss or gain is related with progression of malignancy [65]. However, the biological meaning of Y chromosome instability in tumor progression is still unclear. Interestingly, the introduction of Y chromosome into the human prostate cancer cell line, PC-3, suppressed tumor formation [66], and loss of Yp11.2 containing* TSPY* gene shows strong correlation with tumorigenesis in prostate cancer [67]. And a recent study reported the loss of Y chromosome in the elderly peripheral blood is associated with shorter cancer survival and higher risk of cancer incidence [68]. Since tumor cells have plasticity and phenotypic heterogeneity [69], Y chromosome-loss can be a cause of the phenotypic difference between B7 and H12. Further detailed analyses of Y chromosome-loss, epigenetic status, and transcriptome analysis in H12 are required for better understanding the characteristics of the clone H12 and the mechanism of alveolar differentiation.

**Figure 6:**
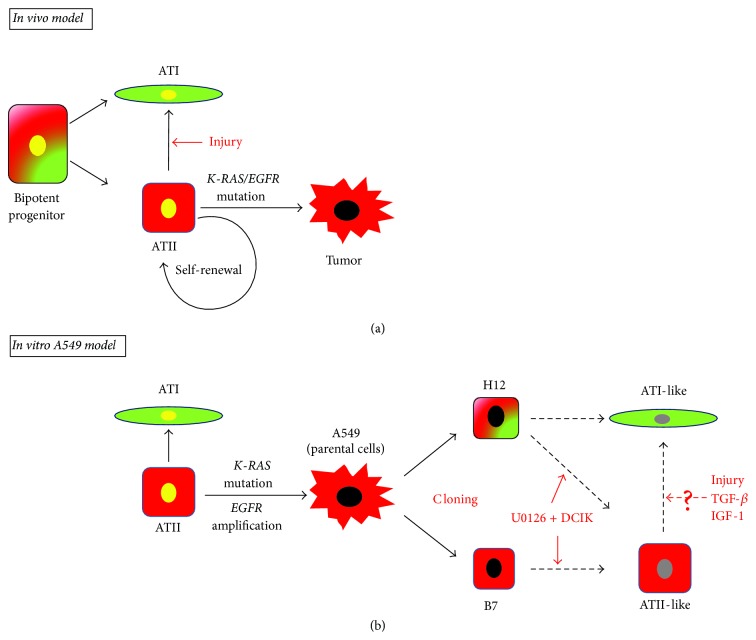
*In vivo* lung alveoli and* in vitro* A549 model. (a)* In vivo* model. Bipotent progenitor cells differentiate into ATI cells and ATII cells. ATII cells have stem cell-like functions, which are able to differentiate into ATI cells by injury and to maintain themselves by self-renewal. When* EGFR* and/or* K-RAS* are mutated, they may cause transformation of ATII cells. (b)* In vitro* A549 model proposed from the current study. A549 has been demonstrated to have* K-RAS* mutation (G12S) and* EGFR* amplification. In A549 clones, B7 have characters of ATII cell, and H12 have characters of both ATI and ATII cells. Both B7 and H12 can be enhanced the cell-type-specific markers of ATII-like cells by treatment of U0126 + DCIK. Injury, TGF-*β*, and IGF-1 may regulate differentiation from ATII-like cells into ATI-like cells. Black arrow: direction of differentiation; red arrow: experimental stimuli; dotted arrow: hypothesis. Yellow oval: nuclei of normal cells; black oval: nuclei of cancer cells; gray oval: nuclei of differentiated clones.

Taken together, we summarized our findings in Figure 6 as an* in vitro* A549 model compared with an* in vivo* model that has been recently reported [25]. During lung alveolar development, the bipotent progenitor cells differentiate into ATI and ATII cells. When alveoli are injured or damaged in adult, ATII cells can differentiate into ATI cells and maintain the supply of ATII cells by self-renewal to keep alveolar homeostasis and restore the tissue defects [21–26]. However, when ATII cells acquired a* K-RAS* mutation and/or* EGFR* mutation, they could be transformed into cancer cells [70, 71].

In an A549 model, A549 has been demonstrated to have* K-RAS* mutation (G12S) and* EGFR* amplification [27–29] and lose some of the characteristics of ATII cells [31, 32]. In our study, we found that A549 clone B7 has characteristics of ATII cells, and H12 has characteristics of both ATI and ATII cells based on the expression of differentiation marker genes. We also observed that ATII-like characteristics could be enhanced or recovered, in both B7 and H12, under the culture condition with U0126 followed by DCIK treatment. Further analyses are required to understand the detailed regulatory mechanisms of ATI and ATII marker gene expression and find out further effective differentiation system using these clones. Our findings suggested that A549 clones would provide a simple and easy* in vitro* model system as the potential representatives of bipotent ATI/II and ATII cells and would elucidate the molecular basis for ATII self-renewal and differentiation from ATII cells into ATI cells.

## 5. Conclusions

In this study, we isolated A549 clones and characterized their distinct characteristics in morphology and gene expression patterns. Among them, we found that two A549 clones, B7 and H12, have ATII cell- and ATI/ATII cell-like characteristics, and they were responsive to the serum depleted stimuli, suggesting that they have the plasticity in gene expression of alveolar differentiation markers. These A549 clones could become the sources of model system to study the molecular basis of regulation for ATII differentiation.

## Figures and Tables

**Table 1:** Primers used for RT-PCR.

Gene	Primer	Annealing (°C)	Cycle	Expressed cells
*FLK *	F: 5′-AAGGCTCAAACCAGACAAGC-3′ R: 5′-TTCCTGCTGGTGGAAAGAAC-3′	58	30	Early mesoderm
*PDGFRα*	F: 5′-GGGGAGAGTGAAGTGAGCTG-3′ R: 5′-AGTCTCGGGATCAGTTGTGC-3′	58	30	

*FOXA2 *	F: 5′-CCGGATCGAGGACAAGTGAG-3′ R: 5′-CAACAACAGCAATGGAGGAG-3′	58	30	Endoderm
*SOX17 *	F: 5′-AAGGGCGAGTCCCGTATCC-3′ R: 5′-TTGTAGTTGGGGTGGTCCTG-3′	58	30	

*TTF-1 *	F: 5′-ACCAGGACACCATGAGGAAC-3′ R: 5′-GCGCCGACAGGTACTTCTG-3′	58	30, 40	Lung progenitor

*p63 *	F: 5′-TTGCCCCTCCTAGTCATTTG-3′ R: 5′-TACTGTCCGAAACTTGCTGC-3′	58	33	Basal

*FOXJ1 *	F: 5′-TGGATCACGGACAACTTCTG-3′ R: 5′-AAGTTGCCTTTGAGGGGTTC-3′	58	40	Ciliated

*MUC5AC *	F: 5′-TACGTGTTCTCCGAGCACTG-3′ R: 5′-GGTTCCACATGAGGACAAGG-3′	58	30	Goblet

*CC10 *	F: 5′-GTCACACTGGCTCTCTGCTG-3′ R: 5′-GAGCAGTTGGGGATCTTCAG-3′	58	40	Clara

*AQP5 *	F: 5′-ATCTTCGCCTCCACTGACTC-3′ R: 5′-TTTCTTCTTTTCCCCCTTGG-3′	56	40	Alveolar type I
*T1α*	F: 5′-TGGGGTCTTACTAGCCATCG-3′ R: 5′-TAGAGGAGCCAAGTCTGGTG-3′	58	40	

*CFTR *	F: 5′-GCCAGCGTTGTCTCCAAAC-3′ R: 5′-CGATAGAGCGTTCCTCCTTG-3′	58	30	Alveolar type II
*SPB *	F: 5′-AAGTTCCTGGAGCAGGAGTG-3′ R: 5′-AGAGGAATGGGGAATTGCTG-3′	58	40	
*SPC1 *	F: 5′-TTGGTCCTTCACCTCTGTCC-3′ R: 5′-CTCCCACAATCACCACGAC-3′	58	40	
*SPC2 *	F: 5′-AACGCCTTCTTATCGTGGTG-3′ R: 5′-AAGACTGGGGATGCTCTCTG-3′	58	35	

*CD133 *	F: 5′-GCAGGGATTATTCTATGCTGTG-3′ R: 5′-ACGCCTTGTCCTTGGTAGTG-3′	58	35	Cancer stem

*HIF-1α*	F: 5′-TGCTCATCAGTTGCCACTTC-3′ R: 5′-TCTCATTTCCTCATGGTCAC-3′	56	30	Cancer

*18SrRNA *	F: 5′-TACCTGGTTGATCCTGCCAGTAGGAT-3′ R: 5′-CCCGTCGGCATGTATTAGCTCTAGAA-3′	58	17	

**Table 2:** Gene expression in A549 parental cells.

Cell type	Early mesoderm		Endoderm		Lung progenitor cells	Basal cells	Ciliated cells	Goblet cells	Clara cells	Alveolar type I cells		Alveolar type II cells		Cancer stem cells
Days of cultures	Marker genes													
	*FLK*	*PDGFRα*	*SOX*17	*FOXA*2	*TTF-1 *	*p*63	*FOXJ*1	*MUC*5*AC*	*CC*10	*T*1*α*	*AQP*5	*SPC*	*CFTR*	*CD*133

d1	—	—	—	+	—	+	+	+	+	—	+	+	—	+
d2	—	—	—	+	—	+	+	+	+	—	—	+	—	+
d3	—	—	—	+	—	+	+	+	+	—	—	+	—	+
d4	—	—	—	+	—	+	+	+	—	—	—	+	—	++

RT-PCR results shown in Figure 1(b) were quantified and normalized by ribosomal 18S. The relative ratio of each gene expression level was evaluated by the lowest expression level among the samples as 1. —: not detected; +: 1–2.5-fold; ++: over 2.5-fold.

**Table 3:** Optical analyses of A549 clones.

A549 clones			Characteristics of cloned cells						
Group number	Plate A	Plate B	Days to confluent	Cell thickness	Cell morphology	Boundary of cell cluster	Cell density	Clusterius	Others

(1)	E2, E5, H9, H10	A2, A3, B2, C6, F5, F9, G1, G2, H10, H11	3.5	Thin	Spindle	Unclear	Low	Low	
(2)		C10, C11	4	Thin	Polygonal	Clear	High	High	
(3)	C5, E8	A8, D12, G5	3.5	Thin	Polygonal	Clear	Low	High	
(4)	G8		14	Thick	Polygonal	Unclear	Low	High	Piling up, granule-rich
(5)		A10, E12	3.5	Thin	Polygonal	Unclear	Low	Low	
(6)	B7, F5	C2, C4, C12, G8	3.5	Thick	Spindle	Unclear	Low	Low	
(7)	F8, H12		4.5	Thick	Polygonal	Clear	High	High	
(8)		B6, B7, B10, C1	4	Thick	Polygonal	Unclear	High	Low	
(9)	A12		3.5	Thick	Polygonal	Unclear	Low	Low	
(10)		D2	3	Thick	Polygonal	Unclear	Low	Low	Granule-rich
(11)	H11	A5, B3, C8, D7, G7	3.5	Thick	Polygonal	Unclear	Low	Low	
(12)	G9-1, G9-2		4.5	Thick	Invadopodia	Unclear	Low	Low	Bright cell body

Optical observations of isolated 46 clones were summarized and categorized into 12 groups. Cell thickness, mean gray value ≥5: thick, <5: thin. Boundary of cell cluster: smoothly draw the outline of cluster: clear; hard to draw the outline: unclear. Cell density, the number of cells in the frame of 100 *μ*m × 100 *μ*m ≥10: high, <10: low. Clustering: the number of cell cluster ≥3: high, <3: low.

**Table 4:** Gene expression patterns in A549 clones.

Cell type	Early mesoderm		Endoderm		Lung progenitor cells	Basal cells	Ciliated cells	Goblet cells	Clara cells	Alveolar type I cells		Alveolar type II cells		Cancer stem cells	Summary of gene expression patterns	Class
A549 clone	*FLK*	*PDGFRα*	*SOX17*	*FOXA2*	*TTF-1*	*p63*	*FOXJ1*	*MUC5AC*	*CC10*	*T1α*	*AQP5*	*SPC*	*CFTR*	*CD133*		
(1) H10	—	—	—	+	—	+	+	++++	+	—	—	—	—	++	Nontypical	1

(2) C11	—	—	—	+	—	+	+	++	+	—	+	+	—	+	ATI ATII	3

(3) C5	—	—	—	+	—	+	—	+	—	—	—	+	—	++	ATII	4

(4) G8	—	+	—	—	—	+	—	—	—	—	—	++	—	—	ATII, no FOXA2 CD133	2

(5) A10	—	—	—	+	—	+	—	+++	—	—	+	+	—	+	ATI ATII	3

(6) C2	—	—	—	+	—	+	++	+++	—	—	—	+	—	+	ATII	4

(7) H12	—	—	—	+	—	+	+++	++++	—	—	++	+	—	++	ATI ATII	3

(8) B7	—	—	—	+	—	+	+++	++	—	—	—	+	—	+	ATII	4

(9) A12	—	—	—	+	—	+	++	+++	—	—	—	+	—	+	ATII	4

(10) D2	—	—	—	+	—	+	+	++++	—	—	+	+	—	+	ATI ATII	3

(11) D7	—	—	—	+	—	+	+	++++	—	—	+	+	—	+	ATI ATII	3

(12) G9	—	—	—	+	—	+	+	++++	—	—	—	+	—	+	ATII	5

RT-PCR results shown in Figure 1(c) were quantified and normalized by ribosomal 18S. The evaluation and indications were the same as in Table 2. —: not detected; +: 1-2-fold; ++: 2-3-fold; +++: 3-4-fold; ++++: 4-5-fold. On the basis of the gene expression patterns and optical features, 12 groups of clones were further categorized into 5 classes of cell characters were selected in this study. Class 1: H10, class 2: G8, class 3: C11, A10, H12, D2, and D7, class 4: C5, C2, B7, and A12, and class 5: G9.
